# RanBP9 at the intersection between cofilin and A*β* pathologies: rescue of neurodegenerative changes by RanBP9 reduction

**DOI:** 10.1038/cddis.2015.37

**Published:** 2015-03-05

**Authors:** J A Woo, T Boggess, C Uhlar, X Wang, H Khan, G Cappos, A Joly-Amado, E De Narvaez, S Majid, L S Minamide, J R Bamburg, D Morgan, E Weeber, D E Kang

**Affiliations:** 1 Department of Molecular Medicine, USF Health Byrd Alzheimer's Institute, Tampa, FL, USA; 2 Department of Molecular Pharmacology and Physiology, USF Health Byrd Alzheimer's Institute, Tampa, FL, USA; 3 Department of Biochemistry and Molecular Biology, Colorado State University, Fort Collins, CO, USA

## Abstract

Molecular pathways underlying the neurotoxicity and production of amyloid *β* protein (A*β*) represent potentially promising therapeutic targets for Alzheimer's disease (AD). We recently found that overexpression of the scaffolding protein RanBP9 increases A*β* production in cell lines and in transgenic mice while promoting cofilin activation and mitochondrial dysfunction. Translocation of cofilin to mitochondria and induction of cofilin–actin pathology require the activation/dephosphorylation of cofilin by Slingshot homolog 1 (SSH1) and cysteine oxidation of cofilin. In this study, we found that endogenous RanBP9 positively regulates SSH1 levels and mediates A*β*-induced translocation of cofilin to mitochondria and induction of cofilin–actin pathology in cultured cells, primary neurons, and *in vivo*. Endogenous level of RanBP9 was also required for A*β*-induced collapse of growth cones in immature neurons (days *in vitro* 9 (DIV9)) and depletion of synaptic proteins in mature neurons (DIV21). *In vivo*, amyloid precursor protein (APP)/presenilin-1 (PS1) mice exhibited 3.5-fold increased RanBP9 levels, and RanBP9 reduction protected against cofilin–actin pathology, synaptic damage, gliosis, and A*β* accumulation associated with APP/PS1 mice. Brains slices derived from APP/PS1 mice showed significantly impaired long-term potentiation (LTP), and RanBP9 reduction significantly enhanced paired pulse facilitation and LTP, as well as partially rescued contextual memory deficits associated with APP/PS1 mice. Therefore, these results underscore the critical importance of endogenous RanBP9 not only in A*β* accumulation but also in mediating the neurotoxic actions of A*β* at the level of synaptic plasticity, mitochondria, and cofilin–actin pathology via control of the SSH1-cofilin pathway *in vivo*.

The defining pathological hallmark of Alzheimer's disease (AD) is the accumulation of amyloid *β* protein (A*β*) in brain associated with tau pathology, synapse loss, cytoskeletal aberrations, mitochondrial dysfunction, and cognitive decline. The generation of A*β* occurs via sequential *β*- and *γ*-secretase processing of the amyloid precursor protein (APP) by beta site APP cleaving enzyme 1 (BACE1) and the presenilin (PS) complex, respectively.^1^ Soluble oligomeric forms of A*β* are thought to be the most toxic species, resulting in synaptic loss and downstream neurotoxicity.^2^ Despite the requirement for Tau in multiple aspects of A*β*-induced neurotoxicity,^3^ a large knowledge gap exists as to how the A*β* oligomer-induced neurotoxic signals are transduced intracellularly to impair synaptic plasticity, eventually leading to neurodegeneration. Both A*β* and Tau promote cofilin–actin pathology,^4, 5^ cofilin–actin pathology is widespread in AD brains,^6^ and cofilin activity is also increased in AD brains.^7^ Cofilin normally functions as a key regulator of actin dynamics that destabilizes filamentous actin (F-actin). Cofilin is inactivated by phosphorylation on Ser3 by LIM kinase 1 (LIMK1), whereas its dephosphorylation by Slingshot homolog 1 (SSH1) activates cofilin.^4^ Upon oxidative stress and/or Ca^2+^ elevation,^4, 8, 9^ SSH1 is activated and active cofilin becomes oxidized on cysteine residues, resulting in rapid mitochondrial translocation to promote apoptosis and induction of cofilin–actin pathology.^10, 11^ An early and consistent impairment secondary to A*β* oligomer treatment in primary neurons is the shrinkage of dendritic spines^12^ involving the rearrangement of F-actin cytoskeleton in spines and loss of spine-associated proteins such as postsynaptic density-95 (PSD95) and Drebrin,^13, 14^ as well as impaired mitochondrial function.^15, 16^

We recently found that overexpression of the scaffolding protein RanBP9 increases A*β* production in cell lines and in transgenic mice.^17, 18^ Moreover, RanBP9 is significantly increased in brains of AD patients and the J20 APP transgenic model.^18, 19^ In studying the trafficking of APP, we also found that RanBP9 overexpression not only promotes the endocytosis of APP but also those of LRP and *β*1-integrin, the latter resulting in disassembly of integrin-associated focal complexes (talin and vinculin).^20^ In addition, RanBP9 overexpression promotes cofilin activation and the translocation of cofilin to mitochondria, resulting in overall mitochondrial dysfunction.^9, 19^ However, how RanBP9 activates cofilin is unknown, and it is not clear whether reduction in endogenous RanBP9 protects against A*β* oligomer-induced deficits in synaptic plasticity, cofilin-dependent pathology, A*β* accumulation, and memory impairment. Here we report that short interfering ribonucleic acid (siRNA) or genetic reduction in RanBP9 significantly reduces SSH1 levels and mitigates A*β*-induced translocation of cofilin to mitochondria, cofilin–actin rod/aggregate formation, depletion of synaptic proteins, deficits in synaptic plasticity, A*β* accumulation, and contextual memory deficits *in vivo*.

## Results

### Endogenous RanBP9 mediates A*β*42 oligomer-induced translocation of cofilin to mitochondria and promotes cofilin activation via positively regulating SSH1

**Figure 1:**
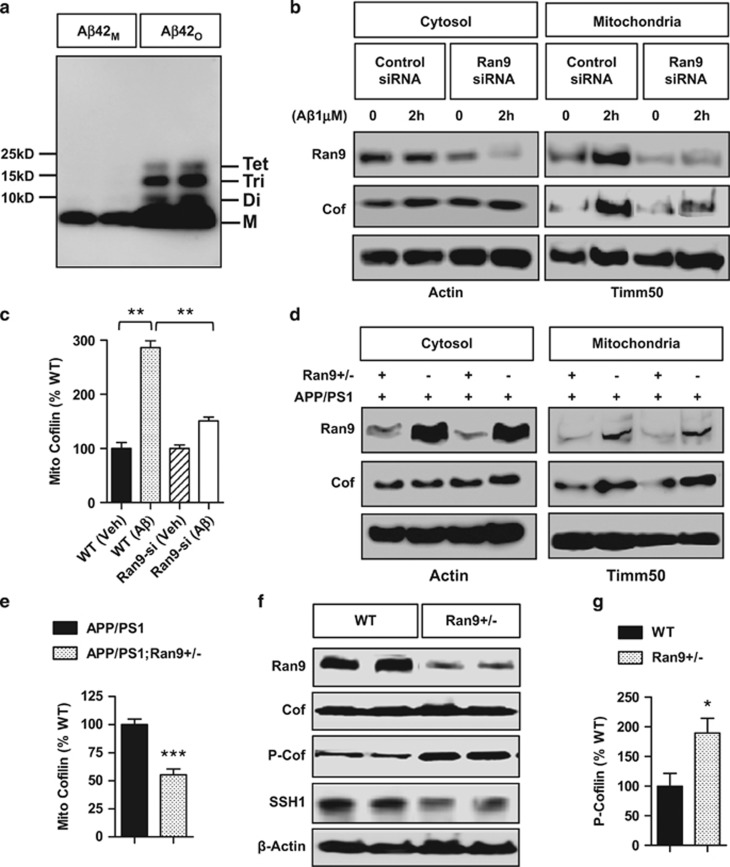

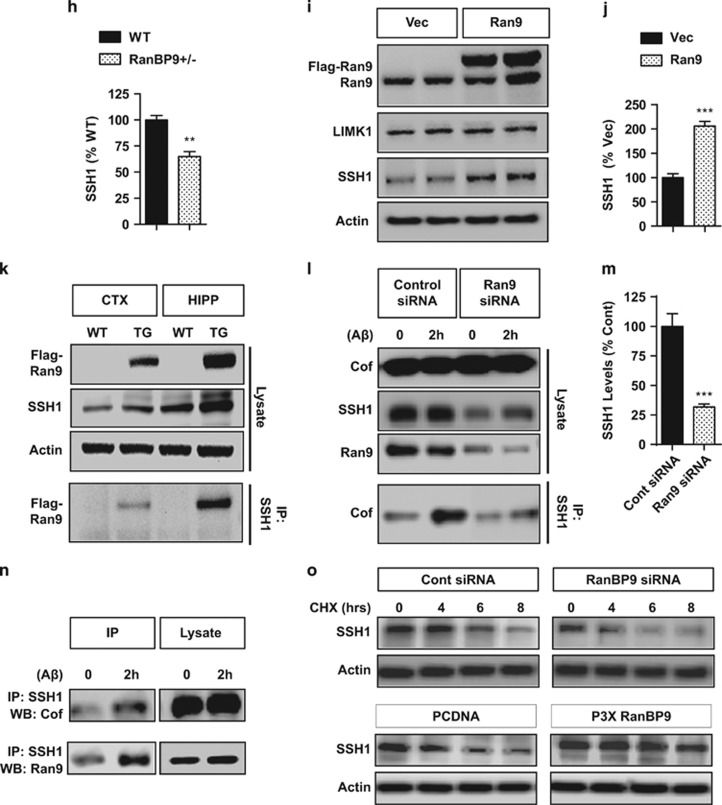
RanBP9 mediates A*β*_O_-induced translocation of cofilin to mitochondria and lowers cofilin activation via SSH1. (**a**) Freshly solubilized A*β*1-42 monomers (A*β*42_M_) or A*β*1-42 oligomer preparation (A*β*42_O_) subjected to SDS-PAGE and immunoblotted for A*β*. Note the monomer (M), dimers (Di), trimmers (Tri), and tetramemers (Tet) in the oligomer preparation. (**b** and **c**) Hippocampus-derived HT22 cells transiently transfected with control or RanBP9 siRNA for 48 h, treated with/without A*β*1-42_O_ for 2 h, separated for mitochondrial and cytosol fractions, and subjected to immunoblotting for the indicated proteins. A representative experiment is shown. Notice the reduction phospho-cofilin upon A*β*42_O_ treatment but mitigated response in RanBP9 siRNA knocked down cells. (**c**) Quantitation of mitochondrial cofilin (ANOVA, post-hoc Tukey, **P<0.005, *n*=3 replicates). (**d** and **e**) Hippocampal extracts from 3-month-old APP/PS1 and APP/PS1;*RanBP9+/−* mice subjected to separation of mitochondrial and cytosol fractions and immunoblotted for the indicated proteins. Note the reduced level of cofilin in mitochondrial fraction of APP/PS1;*RanBP9+/−* brain. (**e**) Quantitation of mitochondrial cofilin (*t*-test, **P<0.005, *n*=4 mice per genotype). (**f**-**h**) Hippocampal extracts from 3-month-old WT and littermate *RanBP9+/−* (Ran9*+/−*) mice subjected to immunoblotting for the indicated proteins. Representative experiment showing increase in phospho-cofilin (*P*-cofilin) and decrease in SSH1 in *RanBP9+/−* brain. (**g**) Quantitation of P-cofilin in hippocampus (*t*=2.74, *P*=0.022, *P<0.05, *n*=5 mice per genotype). (**h**) Quantitation of SSH1 (*t*-test, **P=0.0016, *n*=4 mice per genotype). (**i** and **j**) HT22 cells transiently transfected with vector control (Vec) or Flag-RanBP9 and immunoblotted for the indicated proteins. Note the increase in SSH1 but not LIMK1 by RanBP9 overexpression. (**j**) Quantitation of SSH1 (*t*-test, ***P<0.0001, *n*=4 replicates). Error bars represent S.E.M. on graphs. (**k**) Cortex (CTX) and hippocampus (HIPP) homogenates of 6-month-old WT or littermate Flag-RanBP9 transgenic (TG) mice immunoprecipitated for SSH1 and/or immunoblotted for the indicated proteins. Note the increase in SSH1-RanBP9 complex in Flag-RanBP9 transgenic mice (TG). (**l**) HT22 cells transiently transfected with/without RanBP9 siRNA and treated with/without A*β*42_O_ (1 *μ*M) for 2 h followed by immunoprecipitation for SSH1 and/or immunoblotting for the indicated proteins. Note that RanBP9 siRNA reduces SSH1 levels and decreases A*β*42_O_-induced enhancement of cofilin–SSH1 interaction. (**m**) Quantitation of SSH1 protein levels with/without RanBP9 siRNA transfection in HT22 cells (*t*-test, ***P=0.0008, *n*=4 replicates). (**n**) DIV18 cortical primary neurons treated with or without A*β*42_O_ (1 *μ*M) for 2 h, subjected to immunoprecipitation for SSH1, and/or immunoblotting for the indicated proteins. Note the increase in RanBP9-SSH1 and cofilin-SSH1 complex formation with A*β*42_O_ treatment. (**o**) HT22 cells transiently transfected with control, RanBP9 siRNA, or RanBP9 and subjected to cycloheximide (CHX) treatment for the indicated times followed by immunoblotting for SSH1 or actin. Representative blots adjusted to show similar signal at time 0. Note that RanBP9 siRNA and overexpression accelerates and delays SSH1 turnover, respectively

We assessed whether A*β* oligomers alter cofilin translocation to mitochondria and whether siRNA knockdown of RanBP9 may influence this phenotype. We prepared A*β*1-42 oligomers precisely as previously characterized,^21^ which contained sodium dodecyl sulfate (SDS)-resistant dimers, trimers, and tetramers (A*β*1-42 oligomers (A*β*42_O_)), whereas freshly dissolved A*β*1-42 only contained monomers (A*β*42_M_) (Figure 1a). All stated A*β*42_O_ concentrations heretofore are based on the monomer concentration. Hippocampus-derived HT22 cells were transiently transfected with control or RanBP9 siRNA for 48 h and subjected to treatment with or without A*β*42_O_ (1 *μ*M) for 2 h, followed by separation of intact mitochondria and cytosol fractions. A*β*42_O_ treatment increased both cofilin and RanBP9 translocation to mitochondria (Figure 1b). However, siRNA knockdown of endogenous RanBP9 mitigated A*β*_O_-induced cofilin mitochondrial translocation (Figures 1b and c). A*β*42 monomer exposure (1 *μ*M, 2 h) neither promoted cofilin translocation to mitochondria nor altered cofilin activation (Supplementary Figure S1A). *In vivo*, RanBP9 levels were greatly decreased in the hippocampus of 3-month-old APP/PS1;*RanBP9+/−* mice in both cytosol and mitochondrial fractions compared with APP/PS1 mice^22^ (Figure 1d). Although cofilin levels were unchanged in the cytosol fraction between the genotypes, cofilin was significantly reduced in the hippocampal mitochondrial fraction of APP/PS1;*RanBP9+/−* mice compared with APP/PS1 mice (Figures 1d and e), indicating that endogenous RanBP9 facilitates translocation of cofilin to mitochondria *in vivo*. As expected, 3-month-old *RanBP9+/−* mice exhibited reduced RanBP9 protein together with significantly increased inactive phospho-cofilin without altering total cofilin (Figures 1f and g), which was accompanied by significantly decreased SSH1 protein in *RanBP9+/−* mice (Figures 1f and h), indicating that RanBP9 positively regulates SSH1 levels. Indeed, SSH1 but not LIMK1 levels were significantly increased secondary to RanBP9 overexpression in HT22 cells (Figures 1I and j). Consistent with these observations, RanBP9 not only markedly increased SSH1 but also co-immunoprecipitated with SSH1 in brain (Figure 1k). Accordingly, siRNA knockdown of RanBP9 in HT22 cells strongly reduced the enhancement of cofilin-SSH1 complex induced by A*β*42_O_ treatment (Figure 1l), while significantly decreasing SSH1 levels (Figures 1I and m). In days *in vitro* 21 (DIV21) primary cortical neurons, A*β*42_O_ (2 h) markedly increased RanBP9-SSH1 and cofilin-SSH1 complexes (Figure 1n), suggesting that RanBP9 not only increases SSH1 levels but may also serve to facilitate cofilin-SSH1 interaction upon A*β*42_O_ exposure. To determine whether the RanBP9-SSH1 complex alters SSH1 protein stability, we performed cycloheximide turnover experiments. Indeed, RanBP9 siRNA and overexpression markedly accelerated and delayed the turnover of SSH1, respectively (Figure 1o), confirming that RanBP9 positively regulates SSH1 protein stability.

### Endogenous RanBP9 and SSH1 promote cofilin–actin rod formation and A*β*42 oligomer-induced collapse of growth cones in primary hippocampal neurons

**Figure 2:**
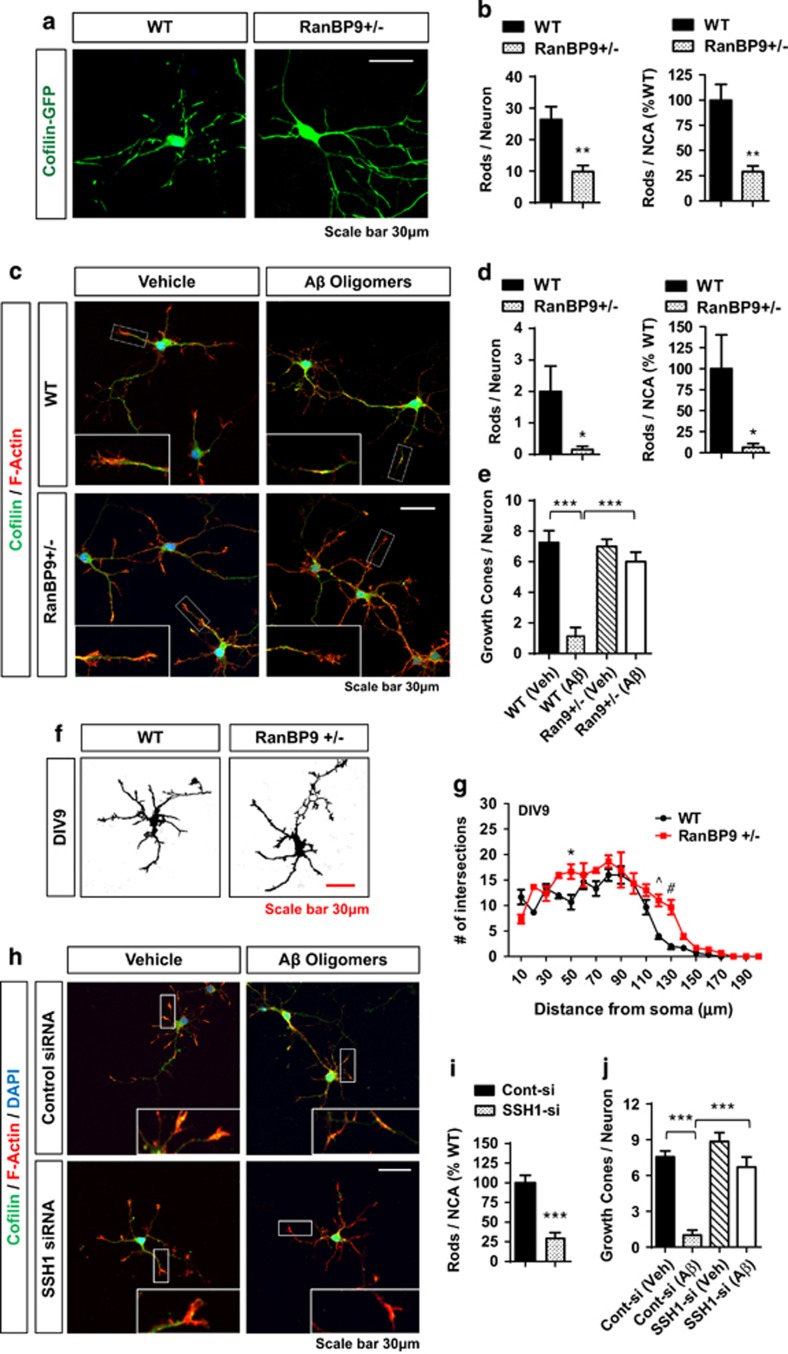
RanBP9 mediates cofilin–actin rod formation and A*β*_O_-induced collapse of growth cones in primary hippocampal neurons. (**a** and **b**) DIV8 hippocampal neurons from WT and *RanBP9+/−* mice transiently transfected with cofilin-GFP and treated with hydrogen peroxide (1 *μ*M) for 30 min and subjected to F-actin staining (Rhodamine-phalloidin). Representative images showing marked reduction in cofilin-GFP rods in *RanBP9+/−* neurons, despite strong GFP-cofilin fluorescence. (**b**) Quantitation of cofilin-GFP rods per neuron (*t*-test, **P=0.0029, *n*=6 replicates from three pups per genotype) or per NCA (*t*-test, **P=0.0009, *n*=6 replicates from three pups per genotype) after treatment of hydrogen peroxide (1 *μ*M) for 30 min. NCA derived from area covered by saturated F-actin stain. (**c**-**e**) DIV8 hippocampal neurons from WT and *RanBP9+/−* mice treated with or without A*β*1-42_O_ for 24 h and subjected to staining for F-actin (Rhodamine-phalloidin) and cofilin. (**c**) Representative images showing no cofilin rods observed without A*β*1-42_O_ treatment but 16.3% and 6.9% of WT and *RanBP9+/−* neurons, respectively, contain cofilin rods. Also note the near absence of F-actin containing growth cones in WT neurons treated with A*β*42_O_ but not in *RanBP9+/−* neurons. (**d**) Quantitation of Cofilin rods per neuron (*t*-test, *P=0.0329, *n*=6 replicates) or per NCA (*t*-test, *P=0.030, *n*=6 replicates from three pups per genotype). (**e**) Quantitation of F-actin containing growth cones in WT and *RanBP9+/−* (Ran9*+/−*) neurons with or without A*β*42_O_ treatment (ANOVA, post-hoc Tukey, ***P<0.0001, *n*=8 replicates from four pups per genotype). (**f** and **g**) Scholl analysis of neurite arborization/elongation in WT and *RanBP9+/−* hippocampal neurons on DIV9. (**f**) Representative images of saturated F-actin stain. (**g**) Quantitation of neurite intersections on concentric circles from the soma in 10*μ*m increments (10–200 *μ*m) (two-way ANOVA, post-hoc Bonferroni, *P<0.05, ^*P*<0.005, ^#^*P*<0.0005, *n*=3 replicates from two mice per genotype). Error bars represent S.E.M. in graphs. (**h**-**j**) DIV8 hippocampal neurons from WT mice with or without control or SSH1 siRNA transfection treated with or without A*β*42_O_ for 24 h and subjected to staining for F-actin (Rhodamine-phalloidin) and cofilin. (**h**) Representative images showing cofilin and F-actin stains. (**i**) Quantitation of cofilin rods per NCA (*t*-test, ***P<0.0001, *n*=6 replicates from three pups per genotype). (**j**) Quantitation of F-actin containing growth cones in neurons transfected with control or SSH1 siRNA with or without A*β*42_O_ treatment (ANOVA, post-hoc Tukey, ***P<0.0001, *n*=8 replicates from four pups per genotype)

Hyperactive cofilin can form cofilin-saturated actin filament bundles (or rods) when challenged by oxidative stress, excitotoxic insult, or A*β* oligomers in primary neurons. These cofilin–actin rods accumulate in neurites, which blocks neuritic transport of key cargo to distal locations.^23, 24^ To assess the role of RanBP9 in cofilin–actin rod formation, we transiently transfected green fluorescent protein (GFP)-cofilin in wild-type (WT) and *RanBP9+/−* hippocampal neurons. On DIV8, we treated neurons with a low concentration of hydrogen peroxide (1 *μ*M) for 30 min, which is known to induce cofilin–actin rod formation. WT neurons formed significantly higher numbers of GFP-cofilin rods per neuron and per neuronal cytoplasmic area (NCA) compared with *RanBP9+/−* neurons (Figures 2a and b). We next treated DIV8 WT and *RanBP9+/−* neurons with or without A*β*42_O_ (1 *μ*M) for 24 h, which has been shown to induce cofilin–actin rod formation in 10–20% of neurons.^25^ We observed fewer percentage of neurons with cofilin rod/aggregate-like structures in *RanBP9+/−* neurons (16.3% of WT neurons and 6.9% of *RanBP9+/−* neurons; Figure 2c), as well as significantly reduced numbers of cofilin rods/aggregates per neuron and per NCA compared with WT neurons upon A*β*42_O_ treatment (Figures 2c and d). In addition, we observed the presence of F-actin-positive growth cones in WT vehicle-treated neurons, which largely collapsed in WT neurons treated with A*β*42_O_ (Figures 2c and e). However, F-actin-positive growth cones were largely unaffected by A*β*42_O_ in *RanBP9+/−* neurons (Figures 2c and e), indicating that RanBP9 reduction protects against cofilin–actin rod formation as well as A*β*42_O_-induced collapse of growth cones. Such changes in *RanBP9+/−* neurons were not due to gross alterations in the numbers or length of neurites (Figures 2f and g). However, Scholl analysis demonstrated that *RanBP9+/−* neurons exhibited subtle but significant increases in numbers of neurite intersections at certain distances from the soma at basal state on DIV9 (Figures 2f and g). Similar to RanBP9 reduction, SSH1 siRNA also significantly prevented cofilin rod/aggregate structures and collapse of growth cones induced by A*β*42_O_ (1 *μ*M) exposure for 24 h (Figures 2h and j), indicating that RanBP9 reduction mitigates A*β*42_O_-induced effects at least in part via negative regulation of SSH1.

### Endogenous RanBP9 mediates the depletion of postsynaptic proteins induced by A*β*42 oligomers in primary hippocampal neurons

**Figure 3:**
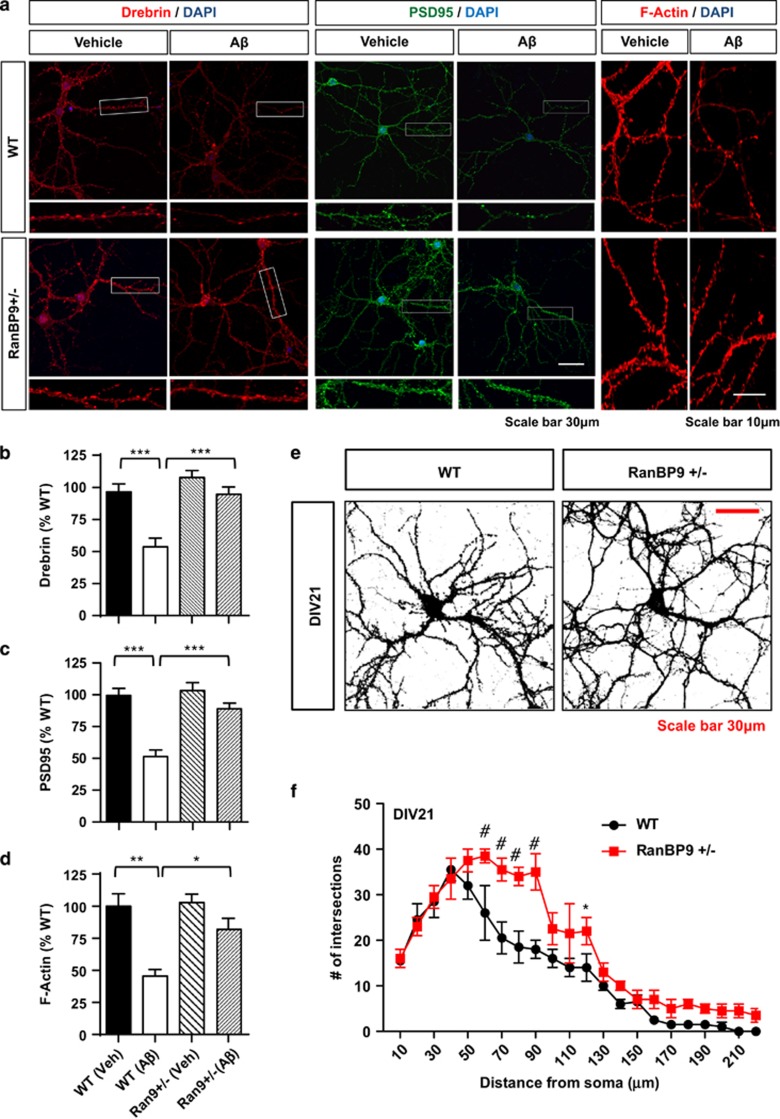
RanBP9 mediates the depletion of postsynaptic proteins and F-actin induced by A*β*1-42_O_ in mature primary hippocampal neurons. (**a**-**d**) DIV21 primary hippocampal neurons derived from P0 *RanBP9+/−* and WT littermate mice treated with or without A*β*1-42_O_ (1 *μ*M) for 2 h and subjected to immunocytochemistry for Drebrin, PSD95, and F-actin (Rhodamine-phalloidin). (**a**) Representative images showing A*β*1-42_O_-induced depletion of Drebrin, PSD95, and F-actin in WT neurons but not in *RanBP9+/−* neurons. (**b**) Quantitation of Drebrin intensity in secondary and tertiary spine-containing dendrites (ANOVA, post-hoc Tukey, ***P<0.0005, *n*=9 replicates from three pups per genotype). (**c**) Quantitation of PSD95 intensity in secondary and tertiary spine-containing dendrites (ANOVA, post-hoc Tukey, ***P<0.0005, *n*=10 replicates from three pups per genotype). (**d**) Quantitation of F-actin (Rhodamine-phalloidin) intensity in secondary and tertiary spine-containing dendrites (ANOVA, post-hoc Tukey, **P<0.005, *P<0.05, *n*=4 replicates from two pups per genotype). Error bars represent S.E.M. on graphs. (**e** and **f**) Scholl analysis of neurite arborization/elongation in WT and *RanBP9+/−* hippocampal neurons on DIV21. (**e**) Representative images of saturated F-actin stain. (**f**) Quantitation of neurite intersections on concentric circles from the soma in 10*μ*m increments (10–220 *μ*m) (two-way ANOVA, post-hoc Bonferroni, *P<0.05, ^#^*P*<0.0005, *n*=3 replicates from two pups per genotype). Error bars represent S.E.M. in graphs

Dendritic spines are structures critical for excitatory synaptic transmission and highly enriched in postsynaptic proteins such as Drebrin, PSD95, and F-actin.^26^ To assess synaptic perturbations induced by A*β*42_O_, we cultured P0 hippocampal neurons derived from *RanBP9+/−* and WT littermate mice. We cultured neurons to DIV21 to visualize mature dendritic spines and treated them with or without A*β*42_O_ for 2 h. As expected, immunocytochemical analysis demonstrated that A*β*42_O_ significantly depleted Drebrin, PSD95, and F-actin in dendritic spines and spine-containing neurites in WT neurons (Figures 3a and d). However, the same A*β*42_O_ treatment had no significant effects in *RanBP9+/−* neurons (Figures 3a and d), indicating that endogenous level of RanBP9 is required for A*β*1-42_O_-induced depletion of postsynaptic proteins and F-actin in mature neurons. A*β*42 monomer treatment (1 *μ*M, 2 h) did not appreciably alter Drebrin or F-actin levels (Supplementary Figure S1B). In DIV21 mature neurons, Scholl analysis demonstrated significantly increased complexity and length of neurites beyond 50 *μ*m from the soma in *RanBP9+/− versus* WT neurons (Figures 3e and f), supportive of a role for endogenous RanBP9 in the inhibition of neurite outgrowth and maturation.

### Significant increase in endogenous RanBP9 in APP/PS1 mice brains

**Figure 4:**
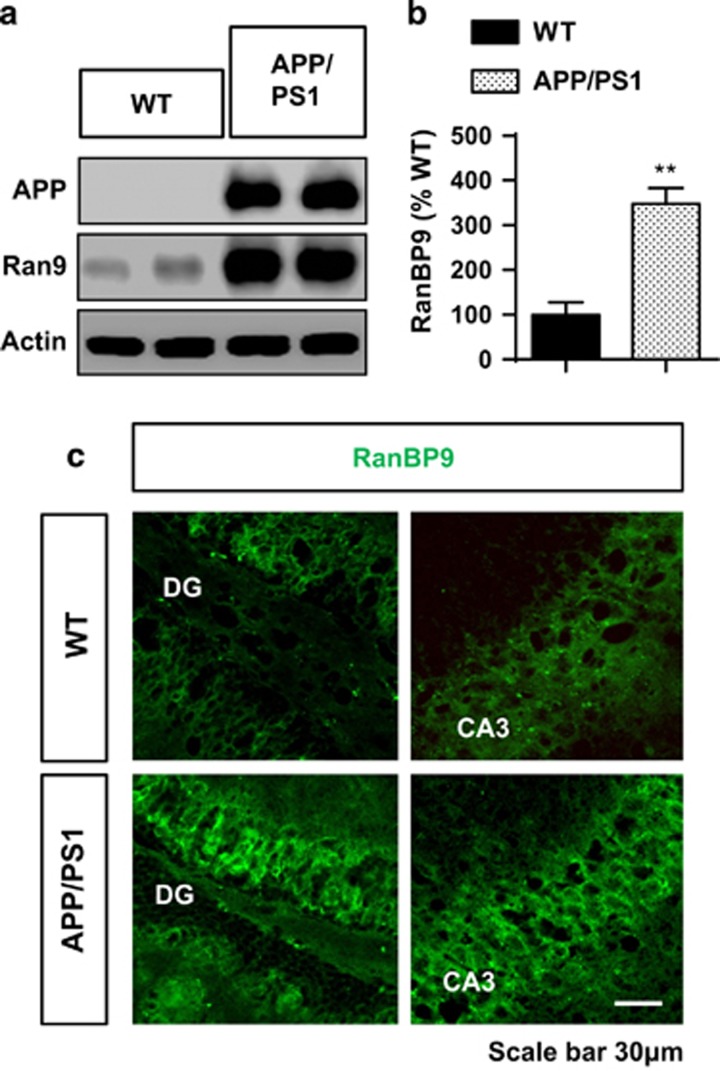
Increased endogenous RanBP9 levels in APP/PS1 mice. (**a** and **b**) Hippocampal extracts from 8-month-old WT and littermate APP/PS1 mice immunoblotted for the indicated proteins. (**a**) Representative blots showing a robust increase in endogenous RanBP9 protein together with overexpression of human APP (6E10 antibody). (**b**) Quantitation of endogenous RanBP9 protein levels in WT and APP/PS1 hippocampus normalized to WT (*t*-test, **P=0.0016, *n*=4 mice per genotype, 2 F and 2 M). (**c**) Representative immunohistochemical images of endogenous RanBP9 immunostaining within the dentate gyrus (DG) and CA3 of the hippocampus

We examined endogenous RanBP9 expression in the APP/PS1 mice expressing the ‘Swedish' and PS1ΔE9 mutation by immunoblotting and immunohistochemistry. Endogenous RanBP9 protein levels were significantly increased by 3.5-fold in the hippocampus of 8-month-old APP/PS1 mice by immunoblotting (Figures 4a and b), consistent with our previous observations in 12-month-old J20 mice harboring the ‘Swedish' mutation and in AD brains.^18, 19^ Further, immunohistochemical analysis of APP/PS1 mouse hippocampus demonstrated greatly enhanced RanBP9 levels in the dentate gyrus (DG) and CA3 regions compared with WT littermates (Figure 4c).

### Rescue of neuroinflammation, synaptic damage, and A*β* accumulation by RanBP9 reduction in APP/PS1 mice

**Figure 5:**
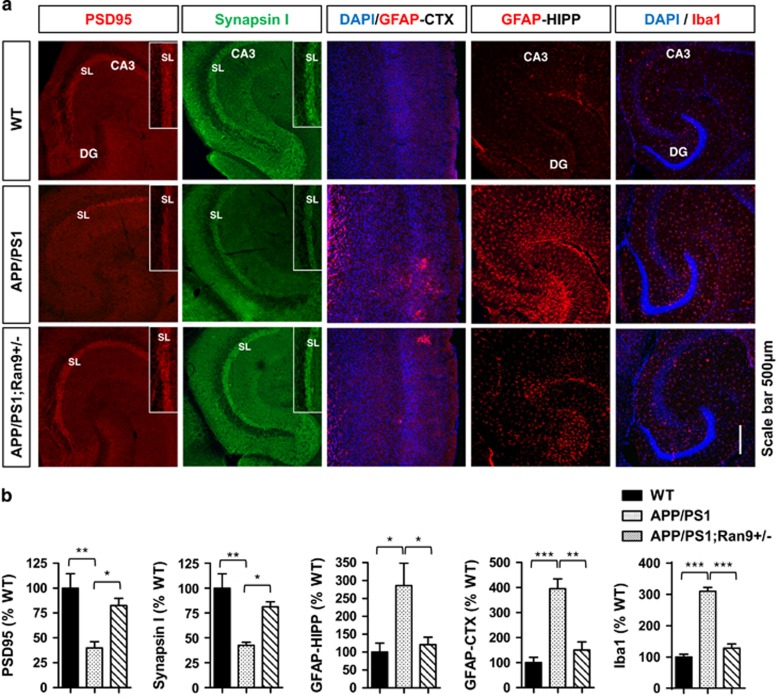
RanBP9 reduction rescues neuroinflammation and synaptic protein depletion in APP/PS1 transgenic mice. (**a**-**e**) Brains from 8-month-old WT, APP/PS1, and APP/PS1;*RanBP9+/−* mice subjected to immunohistochemistry for GFAP (activated astrocyte marker), Iba1 (activated microglia marker), Synapsin I (presynaptic marker), and PSD95 (postsynaptic marker) in the anterior cortex (CTX) and/or hippocampus (HIPP). (**a**) Representative images showing *RanBP9* reduction rescues neuroinflammation (GFAP and Iba1) and synaptic protein loss (Synapsin I and PSD95) in APP/PS1 mice. (**b**) Quantitation of PSD95 intensity within the SL of CA3 (ANOVA, post-hoc Tukey, *P<0.05, **P<0.005, *n*=4 mice per genotype, 2 F and 2 M). Quantitation of synapsin I intensity within the SL of CA3 (ANOVA, post-hoc Tukey, *P<0.05, **P<0.005, *n*=4 mice per genotype, 2 F and 2 M). Quantitation of GFAP intensity in the hippocampus (ANOVA, post-hoc Tukey, *P<0.05, *n*=4 mice per genotype, 2 F and 2 M). Quantitation of GFAP intensity in the anterior cortex (ANOVA, post-hoc Tukey, **P=<0.005, ***P<0.0005, *n*=4 mice per genotype, 2 F and 2 M). Quantification of Iba1 intensity in the hippocampus (ANOVA, post-hoc Tukey, ***P<0.0005, *n*=4 mice per genotype, 2 F and 2 M). Error bars represent S.E.M. on graphs

**Figure 6:**
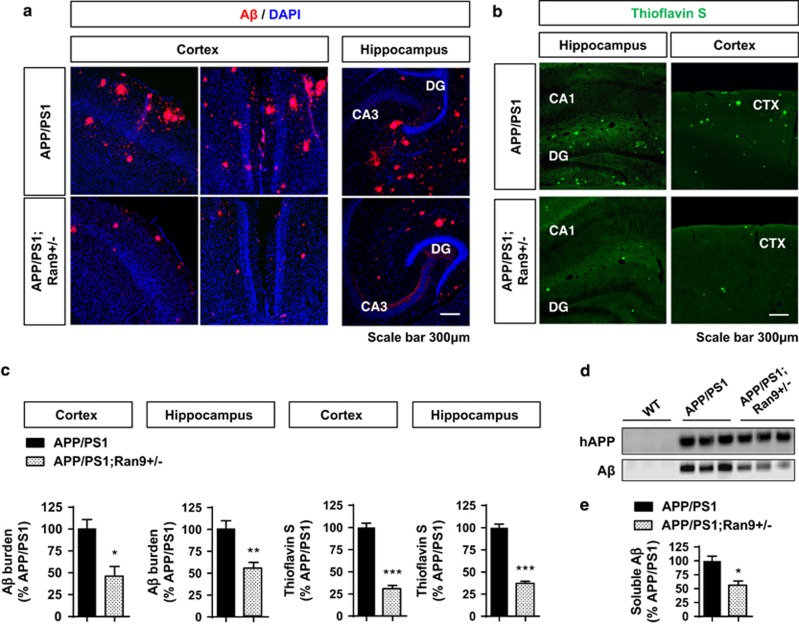
Mitigation of A*β* accumulation by RanBP9 reduction in APP/PS1 mice. (**a**) Representative immunohistochemical images showing reductions in A*β* deposits (red) in the anterior cortex and hippocampus of 8-month-old APP/PS1;*RanBP9+/−* mice compared with littermate APP/PS1 mice. (**b**) Representative images showing reductions in thioflavin S-positive fibrillar A*β* deposits in the anterior cortex and hippocampus of 8-month-old APP/PS1;*RanBP9+/−* mice compared with littermate APP/PS1 mice. (**c**) Quantitation of area covered by A*β* deposits normalized to APP/PS1 mice in the anterior cortex and hippocampus (Cortex: *t*-test,*P=0.0155, *n*=4 each; Hippocampus: *t*-test, **P=0.0067, *n*=4 mice per genotype, 2 F and 2 M). Quantitation of area covered by thioflavin S-positive fibrillary Ab deposits in the anterior cortex and hippocampus (*t*-test, ***P<0.0005, *n*=4 mice per genotype, 2 F and 2 M). Error bars represent S.E.M. in graphs. (**d**) Hippocampal extracts from 6-month-old WT, APP/PS1, and APP/PS1;*RanBP9+/−* mice immunoblotted for human APP (6E10—Triton-X100 extract) and A*β* (PBS extract). (**e**) Quantification of soluble A*β* levels (PBS extract) from 6-month-old APP/PS1 and APP/PS1;*RanBP9+/−* hippocampus (*t*-test, *P=0.0167, *n*=3 mice per genotype)

Given the robust upregulation of RanBP9 levels in APP/PS1 mice, we next determined whether genetic reduction in *RanBP9* can prevent neuroinflammation and synapse loss associated with APP/PS1 transgenic mice. We performed immunohistochemistry for glial fibrillary acidic protein (GFAP) and Iba1 to detect activated astrocytes and microglia as well as Synapsin I and PSD95 to detect pre- and postsynaptic proteins from 8-month-old APP/PS1, APP/PS1;*RanBP9+/−*, and WT littermates. As expected, APP/PS1 mice demonstrated significantly increased GFAP and Iba1 immunoreactivities throughout the hippocampus and areas of the anterior cortex associated with A*β* accumulation as well as diminished PSD95 and Synapsin I immunoreactivities within the stratum lucidium (SL: synaptic terminating zone) of CA3 (Figures 5a and b). In contrast, APP/PS1;*RanBP9+/−* mice showed significantly reduced GFAP and Iba1 immunoreactivities in the hippocampus and anterior cortex, as well as significantly rescued Synapsin I and PSD95 immunoreactivities within the SL of CA3 compared with APP/PS1 littermate mice, essentially indistinguishable from WT mice (Figures 5a and b). As RanBP9 overexpression promotes A*β* production in cultured cells and *in vivo,*^17, 18^ we next examined whether endogenous RanBP9 reduction alters A*β* burden in the hippocampus and anterior cortex of 8-month-old APP/PS1 mice. Indeed, APP/PS1;*RanBP9+/−* mice demonstrated a significant decrease in both the area covered by total A*β* deposits and thioflavin S-positive fibrillar A*β* deposits within the hippocampus and anterior cortex compared with littermate APP/PS1 mice (Figures 6a and c). In addition, soluble A*β* levels were also significantly reduced in the hippocampus of APP/PS1;*RanBP9+/−* mice compared with littermate APP/PS1 mice (Figures 6d and e). These results show that RanBP9 reduction protects against A*β* accumulation, as well as neuroinflammation and synaptotoxicity *in vivo*.

### Accumulation of cofilin–actin rods in APP/PS1 mice and significant attenuation of cofilin–actin rod formation by RanBP9 reduction

**Figure 7:**
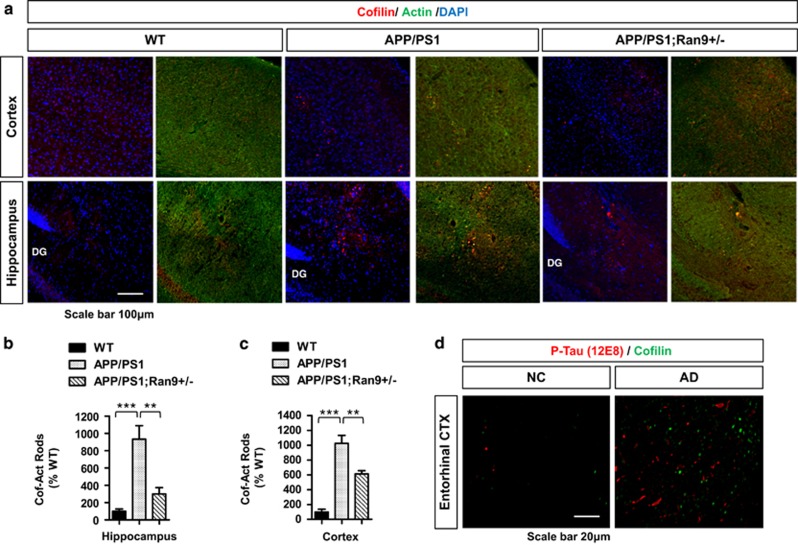
Accumulation of cofilin–actin rod/aggregate structures in APP/PS1 mice resembling those in AD brains and mitigation by RanBP9 reduction. (**a**-**c**) Nine-month-old WT, APP/PS1, and APP/PS1;RanBP9*+/−* littermate brains subjected to immunohistochemistry for cofilin and actin in the anterior cortex and hippocampus using Sudan black to quench background signal. (**a**) Representative images showing increased cofilin–actin rods in APP/PS1 mice and reduction in APP/PS1;*RanBP9+/−* mice. (**b** and **c**) Quantitation of cofilin–actin rods in the hippocampus and anterior cortex normalized to WT (ANOVA, post-hoc Tukey, **P<0.005, ***P<0.0005, *n*=4 mice per genotype). Error bars represent S.E.M. in graphs. (**d**) Representative images of cofilin–actin rods and neuropil threads (phospho-Tau 12E8 antibody) using the same Sudan black quenching immunohistochemical method as in (**a**) in the entorhinal cortex from a cognitively normal control (NC) and AD. Note the marked accumulation of cofilin–actin rods (green) and neuropil threads (red) in AD but few in NC

Given the positive regulation of SSH1 and cofilin, as well as cofilin–actin rods in primary neurons by RanBP9, we assessed cofilin–actin rods/aggregates in WT, APP/PS1, and APP/PS1;*RanBP9+/−* brains. For this, we used Sudan Black B to quench background and the more diffuse non-rod staining. Therefore, only intense signals, such as rods, are visualized, while removing normal cytoplasmic immunoreactivity. Using this method, we found that cofilin rods/aggregates were readily detectable in the hippocampus and anterior cortex of 9-month-old APP/PS1 mice, while very few were found in WT mice (Figure 7a). Such cofilin rod/aggregate structures, which colocalized with actin, were significantly increased by ~10-fold in APP/PS1 *versus* WT mice (Figures 7a and c) and resembled those seen in AD brains (Figure 7d). RanBP9 reduction significantly attenuated cofilin–actin rod/aggregate formation, although not completely to the level of WT mice (Figures 7a and c). In the AD entorhinal cortex, cofilin rod/aggregate structures were numerous and largely did not colocalize with 12E8-positive phospho-Tau immunoreactivity, albeit within the same region and in close proximity (Figure 7d). These findings *in vivo* confirm our findings in primary neurons that RanBP9 reduction ameliorates cofilin-actin pathology in an *in vivo* mouse model of A*β* accumulation.

### RanBP9 reduction rescues deficits in synaptic plasticity and contextual memory associated with APP/PS1 mice

**Figure 8:**
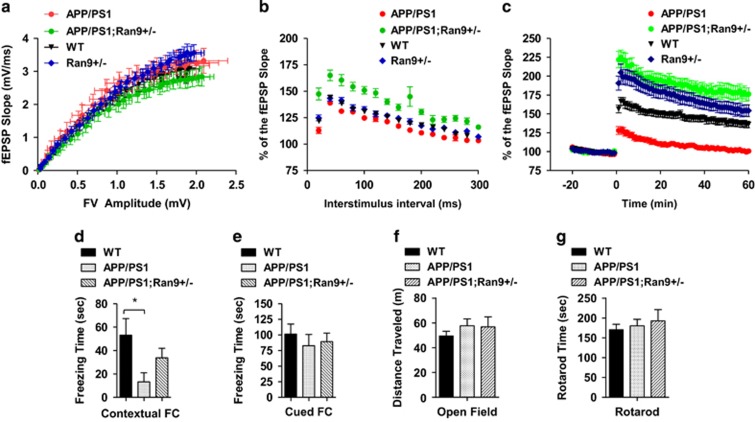
Rescue of synaptic plasticity and contextual memory impairments in APP/PS1 mice by RanBP9 reduction. (**a**-**c**) Stimulating electrode placed in the Schaffer collaterals of the hippocampus and recording glass electrode positioned at the CA1 stratum radiatum below the pyramidal cell layer. (**a**) Input–output analysis performed by stepping up stimulation amplitude from 1–15mV in WT, APP/PS1, *RanBP9+/−*, and APP/PS1;*RanBP9+/−* acute slices. No significant differences observed. Slices from WT *n*=45*, RanBP9+/− n*=31, APP/PS1 *n*=19, APP/PS1;*RanBP9+/− n*=25; slices derived from four to six mice per genotype. (**b**) PPF showing significant differences among all interstimulus intervals (two-way ANOVA, genotype: *P*<0.0001; interstimulus interval: *P*<0.0001; interaction: *P*=0.0122). Post-hoc Tukey test shows significant increases in fEPSP slope in APP/PS1;*RanBP9+/−* slices compared with WT, *RanBP9+/−*, and APP/PS1 slices at nearly all interstimulus intervals (*P*<0.05 to *P*<0.0001). Slices from WT *n*=49, RanBP9*+/− n*=33. APP/PS1 *n*=31, APP/PS1;RanBP9*+/− n*=25; slices derived from four to six mice per genotype. (**c**) No significant changes between genotypes at baseline before LTP but LTP induced by theta burst stimulation showing significant differences in fEPSP slope among WT, APP/PS1, *RanBP9+/−*, and APP/PS1;*RanBP9+/−* slices (two-way ANOVA, genotype: *P*<0.0001; time: *P*<0.0001; interaction: *P*<0.0001). Post-hoc Tukey test shows significant differences between all genotypes at all time points after theta burst stimulation (*P*<0.0001) except between *RanBP9+/− versus* APP/PS1;*RanBP9+/−*. Slices from WT *n*=41, *RanBP9+/− n*=28, APP/PS1 *n*=33, APP/PS1;*RanBP9+/− n*=29; slices derived from four to six mice per genotype. (**d**) Quantitation of contextual fear conditioning (FC) freezing times (sec) after training session 24 h earlier across genotypes. Kruskal–Wallis ANOVA, post-hoc Dunn's, *P<0.05, WT *n*=12 (5 F and 7 M), APP/PS1 *n*=8 (4 F and 4 M), APP/PS1;*RanBP9+/− n*=6 (3 F and 3 M). (**e**) Quantitation of cued fear conditioning (FC) freezing times (sec) across genotypes (no significant differences by Kruskal–Wallis test or one-way ANOVA). Same number and gender as contextual FC. (**f**) Quantitation of open field activity test (total distance traveled over two- day training sessions) across genotypes (no significant differences by Kruskal–Wallis test or one-way ANOVA). Same numbers and gender as contextual FC. (**g**) Quantitation of rotarod test (time staying on rotarod) across genotypes (no significant differences by Kruskal–Wallis test or one-way ANOVA). Error bars represent S.E.M. in graphs. Same number and gender as contextual FC

We tested short-term and long-term synaptic plasticity from acute hippocampal slices prepared from 3-month-old WT, APP/PS1, *RanBP9+/−,* and APP/PS1;*RanBP9+/−* mice, an age when little to no A*β* plaques are detected.^22^ The stimulating electrode was placed in the Schaffer collaterals of the hippocampus, and the recording electrode was positioned at the CA1 stratum radiatum below the pyramidal cell layer. As shown in Figure 8a, input–output analysis did not markedly differ among WT, APP/PS1, and *APP/PS1;RanBP9+/−*, and *RanBP9+/−*slices. However, the APP/PS1;*RanBP9+/−* slices showed a nonsignificant decrease in basal synaptic transmission compared with WT, *RanBP9+/−*, and APP/PS1 slices (Figure 8a). In paired pulse facilitation (PPF) experiments, significant differences were observed in fEPSP slope across genotypes among all interstimulus intervals (Figure 8b). Correction for multiple comparisons surprisingly showed that APP/PS1;*RanBP9+/−* slices exhibited significantly higher PPF compared with both WT, *RanBP9+/−*, and APP/PS1 slices across most interstimulus intervals (Figure 8b), indicating a synergistic effect of RanBP9 and APP/PS1 genotypes on cooperative presynaptic efficacy. For long-term potentiation (LTP) measurements, we detected no differences in fEPSP slope among WT, APP/PS1, *RanBP9+/−*, and APP/PS1;*RanBP9+/−* slices at baseline (Figure 8c). However, after theta burst stimulation, we observed significant differences in fEPSP slope across genotypes for all time points (Figure 8c). Correction for multiple comparisons showed that APP/PS1 slices were significantly impaired in fEPSP slope compared with WT, *RanBP9+/−*, and APP/PS1;*RanBP9+/−* slices at every time point up to 1 h (Figure 8c). APP/PS1;*RanBP9+/−* slices exhibited significantly stronger fEPSP slope across all time points compared with WT and APP/PS1 slices (Figure 8c), indicating that *RanBP9* reduction greatly enhances both the induction and maintenance of LTP. Similarly, *RanBP9+/−* slices exhibited significantly stronger LTP than WT slices up to 30 min and did not significantly differ from APP/PS1;*RanBP9+/−* slices (Figure 8c). Therefore, these results indicate that RanBP9 reduction not only rescues the deficits in synaptic plasticity associated with the APP/PS1 mice but also further potentiates synaptic plasticity.

To determine whether the changes in LTP and synaptic proteins correlate with learning and memory, we carried out fear conditioning tests (contextual and cued), in which WT, APP/PS1, and APP/PS1;*RanBP9+/−* mice were trained on day 1 for both. On day 2, we tested mice for contextual and cued conditioning memory. As shown in Figure 8d, we observed significant differences across genotypes in the amount of time spent freezing (in seconds) in the contextual fear conditioning test (Figure 8d). Specifically, WT mice froze for significantly longer than APP/PS1 mice but did not significantly differ from the APP/PS1;*RanBP9+/−* mice (Figure 8d), as the latter mice showed an intermediate amount of time freezing. However, we did not observe significant differences across genotypes in the hippocampus-independent cued conditioning freezing times (Figure 8e). We also did not find significant differences across the three genotypes in open field or rotarod tests (Figures 8f and g), indicating the these genotypes do not alter general locomoter activity or coordination. Taken together, these results show that a threshold of endogenous RanBP9 is required to mediate the full deficits in hippocampus-dependent contextual memory but not cued memory.

## Discussion

Molecular pathways that govern the neurotoxicity and production of A*β* are attractive therapeutic targets for AD. In this study, we utilized HT22 cells, primary neurons, acute brain slices, and genetically modified mice to gain insights into the role of endogenous RanBP9 in A*β*-induced synaptic degeneration, cofilin activation, cofilin–actin rod formation, and A*β* accumulation in a mouse model of AD. We made a number of novel observations demonstrating that endogenous RanBP9 positively regulates SSH1 levels and is critical for A*β*-induced translocation of cofilin to mitochondria, cofilin–actin rod/aggregate formation, collapse of growth cones, loss of synaptic proteins, and impairments in synaptic plasticity and learning/memory. Our findings underscore the critical importance of the RanBP9–SSH1–cofilin pathway in AD pathogenesis.

A prerequisite for cofilin translocation to mitochondria and formation of cofilin–actin rods is its activation via dephosphorylation and oxidation on several cysteine residues.^10, 11^ Therefore, the observation that A*β* oligomers induced cofilin translocation to mitochondria and formation of cofilin–actin rods infers its rapid activation and oxidation by A*β* oligomers. Interestingly, A*β*_O_ also increased RanBP9 translocation to mitochondria, which may in part be explained by its association with the pro-apoptotic mitochondria-associated protein P73.^27^ Genetic reduction and siRNA knockdown of RanBP9 significantly mitigated both of these measures and reduced cofilin activation. As cofilin is activated by SSH1-dependent dephosphorylation and inactivated by LIMK-dependent phosphorylation, it is plausible that RanBP9 alters one or both of these pathways. Indeed, we found that RanBP9 positively regulates SSH1 but not LIMK1 levels in HT22 cells and in brain, which accounts for increased cofilin activation by RanBP9. As RanBP9 levels are robustly increased in APP/PS1 mouse brains, it is likely that A*β*_O_ also activates cofilin via a mechanism involving RanBP9 and SSH1. A*β* is known to increase cytosolic Ca^2+^ and activate calcineurin as well as enhance production of reactive oxygen species (ROS),^28, 29^ both of which are known activators of SSH1. Calcineurin-dependent dephosphorylation of SSH1 serves to activate SSH1,^30^ and cysteine oxidation of 14-3-3*ζ/τ* removes the inhibition of SSH1 by 14-3-3*ζ/τ*.^8^ In addition, cysteine oxidation of cofilin itself is required for both mitochondrial translocation and cofilin–actin rod formation.^10, 11^ We recently showed that A*β*_O_ promotes cofilin–actin rod formation via a pathway requiring PrP^c^ and NADPH oxidase (NOX), the latter which is involved in the generation of ROS.^31^ We have also shown that A*β*_O_ and RanBP9 impair mitochondrial Ca^2+^ buffering and promote mitochondrial superoxide production in a cofilin-dependent manner.^9, 19^ Therefore, the engagement of A*β*_O_ to its neuronal receptors likely involves downstream Ca^2+^ and ROS from multiple sources. Activated/oxidized cofilin likely contributes to sustaining this cycle by promoting mitochondrial superoxide production.

We observed that *RanBP9+/−* neurons are resistant to A*β* oligomer-induced collapse of F-actin containing growth cones in immature DIV9 neurons and depletion of postsynaptic proteins (Drebrin, PSD95, and F-actin) in mature DIV21 neurons. Such findings are likely explained, at least in part, by the reduction in cofilin activation status in *RanBP9+/−* neurons. Specifically, phosphorylation and dephosphorylation of cofilin are associated with dendritic spine growth and shrinkage during LTP and LTD, respectively,^32, 33, 34^ and cofilin regulates the trafficking of *α*-amino-3-hydroxy-5-methyl-4-isoxazolepropionic acid (AMPA) receptors in dendritic spines.^35^ Further, *β*-arrestin 2 recruits cofilin to dendritic spines upon *N*-methyl-D-aspartate receptor activation to control the remodeling of spines, and *β*-arrestin 2 knockout neurons are resistant to A*β*-induced dendritic spine loss through spatial control of cofilin activation.^36^

Despite the crucial involvement of cofilin in synaptic plasticity, the aforementioned mechanism alone is unlikely to explain the full extent of the rescue and enhancement of LTP and PPF associated with APP/PS1;*RanBP9+/−* mice, as both LTP and PPF in were strongly enhanced significantly beyond those of WT slices. However, in the absence of the APP/PS1 genotype, *RanBP9+/−* mice exhibited normal PPF but significantly enhanced LTP. The exaggerated PPF in APP/PS1;*RanBP9+/−* slices may be explained by lower probability of Ca^2+^-evoked neurotransmitter release as evidenced by somewhat reduced fEPSP slope per stimulus amplitude (input/output curve). However, the second paired Ca^2+^ signal appears to overcompensate to release a larger pool of neurotransmitter. We hypothesize that RanBP9 reduction enhances synaptic associativity and cooperativity perhaps at the level of synapse formation and maintenance. Indeed, *RanBP9+/−* slices produced significantly enhanced LTP compared with WT and did not differ significantly from APP/PS1;*RanBP9+/−* slices. Overexpression of RanBP9 significantly reduces axon elongation in the chick spinal cord via Plexin-A,^37^ and we also showed in this study that reduction in *RanBP9* increases neurite arborization/complexity in mature neurons and protects against both A*β* oligomer-induced growth cone collapse and dendritic postsynaptic protein depletion. Previous studies have also shown the involvement of RanBP9 in negatively regulating *β*1-integrin function^20^ and a role in the nucleocytoplasmic regulation of cell morphology via its interaction with muskelin.^38^ Therefore, RanBP9 may be involved in regulating neuritogenesis, synaptic maintenance, and synaptic plasticity changes via these pathways in addition to cofilin activation.

RanBP9 reduction protected against both A*β*_O_- and hydrogen peroxide-induced cofilin–actin rod formation in primary neurons, thereby indicating a direct effect of RanBP9 and SSH1 reduction on cofilin–actin rods. Indeed, SSH1 reduction *per se* significantly mitigated A*β*42_O_-induced cofilin rod/aggregate formation and collapse of growth cones, indicating that SSH1 reduction, at least in part, is responsible for such effects of RanBP9. *In vivo*, however, RanBP9 reduction mitigated both cofilin–actin rod/aggreagate formation and A*β* accumulation/plaques. Therefore, it is likely that RanBP9 reduction lowers cofilin–actin rod/aggregate formation both directly via SSH1 regulation and indirectly by reducing A*β* accumulation. Cofilin–actin rods are thought to be an initial protective response to oxidative insults;^39^ however, persistent accumulation of rods physically blocks neuritic transport of cargo and impairs synaptic activity.^23, 24^ Inhibition of RanBP9 and SSH1 may not be sufficient to fully block cofilin–actin pathology, as ROS pathways acting directly on cofilin may also promote cofilin–actin pathology. In this light, it is intriguing that Tau overexpression induces cofilin–actin pathology and potently increases F-actin bundling,^5^ while disrupting mitochondrial fission via an actin-dependent mechanism.^40^ Taken together, this study demonstrated the crucial involvement of endogenous RanBP9 in A*β*-induced cofilin activation via SSH1 and downstream consequences on cofilin–actin rod formation, A*β* accumulation, synaptic plasticity, and contextual memory. These findings underscore the role of RanBP9 and its associated molecular pathway as critical mediators of AD pathology and potential therapeutic targets.

## Materials and Methods

### Cells, complementary deoxyribonucleic acid constructs, siRNA sequences, and transfections

Hippocampus-derived HT22 cells were maintained in Dulbecco's modified Eagle's medium containing 10% FBS. HT22 cells were obtained from Professor David Schubert (Salk Institute, San Diego, CA, USA). The RNAi targeting RanBP9 (5′-UCUUAUCAACAAUACCUGC-3′) and SSH1 (5′-GAGGAGCUGUCCCGAUGAC-3′) were obtained from GE Dharmacon (Lafayette, CO, USA). All cells were transfected for 48 h using lipofectamine 2000 (Invitrogen, Carlsbad, CA, USA) according to the manufacturer's instructions with siRNA duplexes or control single-stranded denatured control siRNAs before performing biochemical and/or immunocytochemical assays. All siRNAs were transfected at a final concentration of 100 nM. Cortical and hippocampal primary neurons were derived from postnatal day 0 (P0) pups and grown on poly-D-lysine-coated coverslips or plates as previously described.^19^ Quantitation of digitized immunoblotting data was performed using the Image J software (NIH Image J, Bethesda, MD, USA).

### Antibodies, reagents, and A*β* oligomer preparation

Antibodies to APP (6E10, Covance, Madison, WI, USA), cofilin (D3F9, Cell Signaling, Danvers, MA, USA), phospho-cofilin (77G2, Cell Signaling), actin (AC-74, Sigma Aldrich, St. Louis, MO, USA), tubulin (TU-02, Santa Cruz Biotechnology, Dallas, TX, USA), A*β* (D54D2, Cell Signaling), GFAP (Invitrogen), synapsin I (Invitrogen), PSD95 (Abcam, Cambridge, MA, USA), Drebrin (Abcam), microtubule-associated protein 2 (Millipore, Billerica, MA, USA), HRP-linked secondary antibodies (Jackson Immunochemicals, West Grove, PA, USA), and fluorescently labeled secondary antibodies (Invitrogen) were obtained from the indicated sources. The mouse monoclonal anti-RanBP9 antibody was a generous gift from Professor Elizabetta Bianchi (Pasteur Institute, France). Synthetic A*β*1-42 peptide was purchased from American Peptide (Sunnyvale, CA, USA). A*β*1-42 oligomers were prepared as previously characterized.^21^ Briefly, A*β*1-42 powder was dissolved in hexafluoro-2-propanol (HFIP) at 1 mM for 30 min at room temperature, aliquoted to eppendorf tubes, allowed to evaporate overnight in fume hood, and subjected to speed vacuum for 1 h to remove traces of HFIP or moisture. To prepare A*β* oligomers, A*β*1-42 film was then dissolved in dimethyl sulfoxide (5 mM), and F-12 cell culture medium (without phenol) was added to a final concentration of 100 *μ*M A*β*1-42 and incubated at 4 °C for 24 h.

### Cell/tissue lysis and immunoblotting

Cultured cells or brain homogenates were lysed with lysis buffer (50 mM Tris-Cl, 150 mM NaCl, 2 mM ethlenediaminetetraacetic acid, and 1% TritonX-100). For isolation of mitochondria, mitochondrial isolation kit (Thermo Scientific, Rockford, IL, USA) was used according to the manufacturer's instructions. Protein quantification was performed by a colorimetric detection reagent (BCA Protein Assay, Pierce, Rockford, IL, USA). Equal amounts of protein were subjected to SDS-polyacrylamide gel electrophoresis (PAGE) and transferred to nitrocellulose membranes for immunoblotting. After probing with the primary antibody, the corresponding peroxidase-conjugated secondary antibody was detected by electrochemiluminescence (ECL) western blot reagents (Pierce).

### Mice

APP/PS1, WT, and *RanBP9+/−* mice^19^ were all bred in the C57BL6 background for at least three generations before interbreeding with each other. APP/PS1 mice, which express ‘Swedish' APP and PS1 ΔE9 mutations, were obtained from Jackson Immunoresearch (West Grove, PA, USA).^22^

### Immunofluorescence

Immunohistochemistry and immunocytochemistry were performed as previously described.^19^ Briefly, animals were perfused with 4% paraformaldehyde in PBS, and brains were post-fixed in the same fixative for 24 h. The brains were then cryoprotected in 30% sucrose and sectioned (30 *μ*m) on a cryostat or microtome. For primary neurons and HT22 cells, cells were fixed in 4% paraformaldehyde for 15 min at room temperature. After blocking with normal goat serum, primary antibodies were applied overnight at 4 °C, and secondary antibodies were applied for 45 min at room temperature, followed by counterstaining with Hoechst33342 or DAPI and mounting. For detection of cofilin–actin rods in brain, 70% ethanol was applied to sections for 5 min after washing the secondary antibody, followed by 0.1% Sudan Black B in 70% ethanol for 12 min, two washes with 70% ethanol, several washes in Tris-buffered saline (TBS), and rinsed in H_2_0 before mounting. All immunoreactivities were quantitated from every 12th serial section through an entire hippocampus or anterior cortex. All images were acquired with the Olympus FV10i confocal microscope (Tokyo, Japan) and quantitated using the Image J software. Comparison images were acquired with identical laser intensity, exposure time, and filter. Adjustments to the brightness/contrast were applied equally to all comparison images.

### Behavioral analysis

Fear conditioning (contextual and cued), rotarod, and open field tasks were performed as previously described.^41^ For fear conditioning, an aversive stimulus (in this case a mild foot shock, 0.5 mA) was paired with an auditory conditioned stimulus (white noise) within a novel environment. Training consisted of two mild shocks paired with two conditioned stimuli with a 3-min interval between each shock. Freezing on the training day in response to the foot shock was used as an estimate of learning during the acquisition trial. To test conditioning to the context, animals were re-introduced to the same training chamber for 6 min and freezing behavior was recording by tracking software (Any-maze, Wood Dale, IL, USA) every second. To test conditioning to the tone, animals were introduced to a novel context, consisting of a chamber with different shape, floor and olfactory cues from the training chamber. Mice were scored for 3 min, before and after the tone in the same manner described above. Learning was assessed by measuring freezing behavior (i.e., motionless position) every second. The open field was used as a standard test of general activity. Briefly, animals were monitored for 15 min in a 40 cm square open field with a video tracking software (Any-maze). General activity levels were evaluated by measurements of total distance traveled. Motor performance was evaluated by an accelerating rotarod apparatus with a 3 cm diameter rod starting at an initial rotation of 4 r.p.m. slowly accelerating to 40 r.p.m. over 5 min. The time spent on the rod during each of four trials per day for 2 consecutive days was measured previously described in.^42^

### Electrophysiology

Hippocampus slices were prepared from 3-month-old WT, APP/PS1, and APP/PS1;*RanBP9+/−* mice and subjected to input/output curved, paired pulse facilitation, and LTP as previously described.^43^ Briefly, animals were killed, brains were harvested, and sectioned horizontally (400 *μ*m) in ice-cold cutting solution (110 mM sucrose, 6 mM NaCl, 3 mM KCl, 26 mM NaHCO_3_, 1.25 mM NaH_2_PO_4_, 7 mM MgCl_2_, 0.5 mM CaCl_2_, 10 g/l glucose, pH 7.3–7.4). The hippocampus was dissected and acclimated in 50:50 solution (cutting:artificial cerebrospinal fluid (ACSF)) for 10 min at room temperature. Then the slices were transferred to ACSF (125 mM NaCl, 2.5 mM KCl, 1.25 mM NaH_2_PO_4_, 0.26 mM NaHCO_3_, 1.2 mM MgCl_2_, 2.0 mM CaCl_2_, and 10 g/l glucose, pH 7.3–7.4, saturated with 95% O_2_ and 5% CO_2_). Slices were recovered in ACSF at room temperature at least 40 min, followed by a final incubation in ACSF for 1 h at 30 °C.

Extracellular field potential recording, LTP: the recording chamber was held at 30±0.5 °C with ACSF flow rate of 1 ml/min. The stimulating electrode was placed in the Schaffer collaterals of the hippocampus. The recording glass electrode loaded with ACSF was positioned at the CA1 stratum radiatum below the pyramidal cell layer. Stimulating pulses were generated by the Digidata 1322A interface (Molecular Devices, Sunnyvale, CA, USA) and a stimulus isolator (model 2200; A-M Systems, Sequim, WA, USA) under control of Clampex 10.0 software (Molecular Devices). Field excitatory postsynaptic potentials (fEPSP) were amplified using a differential amplifier (model 1800; A-M Systems), filtered at 1 kHz, and digitized at 10 kHz.

Input–output analysis was performed by stepping stimulation amplitude from 1 to 15mV. Stimulation amplitude that elicited half-maximal fEPSP was determined by the input–output curve and stimulation rate of 0.05 Hz was used through the whole experiment. PPF, which is short-term plasticity, was evoked by two pulses with interpulse intervals from 20 to 300 ms. Percentage of the facilitation was calculated by dividing fEPSP slope elicited by the second pulse with the fEPSP slope elicited by the first pulse. LTP was induced by theta burst stimulation (TBS) (five trains of four pulses at 200 Hz separated by 200 ms, repeated six times with an inter-train interval of 10 s). LTP was sampled 60 min after the induction, and calculated by dividing the slope of 60 min post-induction responses with the average slope of 20-min baseline responses.

### Statistical analysis and graphs

Statistical data were analyzed by the GraphPad Prizm 6.0 software (GraphPad Software, San Diego, CA, USA) using Student's *t*-test, one-way or two-way analysis of variance (ANOVA). ANOVA was followed by Tukey or Bonferroni post-hoc tests. In tests with high variance and lack of normal distribution, Kruskal–Wallis statistic was employed, followed by Dunn's post-hoc analysis. All quantitative graphs were expressed as mean±S.E.M. Differences were deemed significant when *P*<0.05.

## Abbreviations

A*β*: amyloid *β* protein
A*β*42_M_: amyloid *β* protein 1-42 monomers
A*β*42_O_: amyloid *β* protein 1-42 oligomers
ACSF: artificial cerebrospinal fluid
AD: Alzheimer's disease
ANOVA: analysis of variance
APP: amyloid precursor protein
DIV: days *in vitro*
F-Actin: Filamentous Actin
fEPSP: field excitatory postsynaptic potential
GFAP: glial fibrillary acidic protein
GFP: green fluorescent protein
HFIP: Hexafluoro-2-propanol
LIMK1: LIM Kinase 1
LTP: long-term potentiation
NCA: neuronal cytoplasmic area
PAGE: polyacrylamide gel electrophoresis
PPF: paired pulse facilitation
PS1: presenilin-1
PSD95: postsynaptic density-95
SDS: sodium dodecyl sulfate
siRNA: short interfering ribonucleic acid
SL: stratum lucidum
SSH1: Slingshot homolog 1
TBS: Tris-buffered saline
WT: wild type
