# Supplementary material for: RanBP9 at the intersection between cofilin and Aβ pathologies: rescue of neurodegenerative changes by RanBP9 reduction
Source: Cell Death Dis. 2015 Mar 5;6(3):1676–. doi: 10.1038/cddis.2015.37 (PMC4385917; doi:10.1038/cddis.2015.37)
Supplement: Supplementary Figure 1 [file cddis201537x1.ppt]

## Slide 1
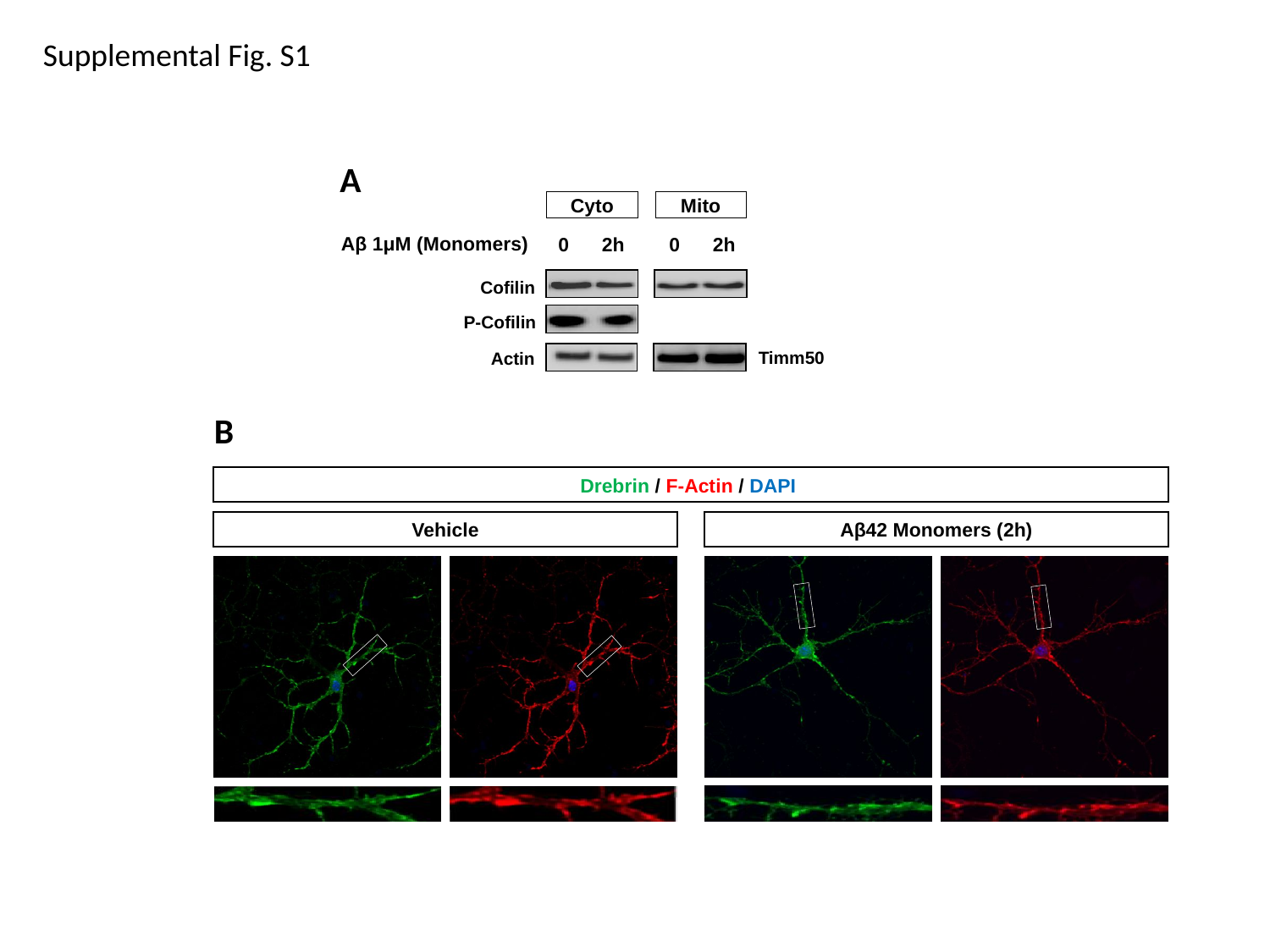

Supplemental Fig. S1
A
Cyto
Mito
 Aβ 1μM (Monomers)
 0 2h
 0 2h
 Cofilin
 P-Cofilin
 Timm50
 Actin
B
Drebrin / F-Actin / DAPI
Vehicle
Aβ42 Monomers (2h)
