# Supplementary material for: RanBP9 at the intersection between cofilin and Aβ pathologies: rescue of neurodegenerative changes by RanBP9 reduction
Source: Cell Death Dis. 2015 Mar 5;6(3):1676–. doi: 10.1038/cddis.2015.37 (PMC4385917; doi:10.1038/cddis.2015.37)
Supplement: Supplementary Figure Legends [file cddis201537x2.doc]

**Supplemental Figure Legends**

**Supplemental Figure S1. Short-term Aβ42 Monomer Exposure Does Not Alter Cofilin Translocation to Mitochondria or Drebrin/F-Actin Levels**

(A) Hippocampus-derived HT22 cells treated with/without Aβ42 monomers for 2h, separated for mitochondrial and cytosol fractions, and subjected to immunoblotting for the indicated proteins. A representative experiment shows no effect of Aβ42 monomers in Cofilin translocation to mitochondria or activation status.

(B) DIV21 primary hippocampal neurons derived from P0 *RanBP9+/-* and wild type (WT) littermate mice treated with or without Aβ42 monomers (1μM) for 2h and subjected to immunocytochemistry for Drebrin and F-Actin (Rhodamine-phalloidin). Representative images show no effect of Aβ42 monomers on Drebrin or F-Actin levels.
